# Draft Genome Sequences of 23 *Tenacibaculum* Isolates from Farmed Norwegian Lumpfish, *Cyclopterus lumpus*

**DOI:** 10.1128/mra.01249-21

**Published:** 2022-03-15

**Authors:** Bjørn Spilsberg, Hanne K. Nilsen, Snorre Gulla, Karin Lagesen, Anne Berit Olsen, Duncan J. Colquhoun

**Affiliations:** a Norwegian Veterinary Institute Ås, Ås, Norway; b Norwegian Veterinary Institute Bergen, Bergen, Norway; Montana State University

## Abstract

Draft genome sequences of 23 *Tenacibaculum* sp. strains that were isolated from Cyclopterus lumpus (lumpfish) were investigated to elucidate possible routes of transmission between Salmo salar (Atlantic salmon) and lumpfish.

## ANNOUNCEMENT

The salmon louse (Lepeophtheirus salmonis) is the most common parasite in Norwegian salmon farming at sea and constitutes a serious welfare threat to the Atlantic salmon itself and to wild salmonid populations (1). Cleaner fish is one option to combat salmon lice in fish farms. However, both cleaner fish, like lumpfish, and salmon are susceptible to infections by *Tenacibaculum* spp. (2), and it can be speculated that infected lumpfish can spread the infection to salmon and vice versa. Bacterial swab samples from skin ulcers, kidney, and/or spleen from lumpfish that were used as cleaner fish in salmon farming were plated on marine agar (Difco) and incubated at 15°C for up to 7 days. Round-to-ovoid, yellow-pigmented colonies morphologically consistent with *Tenacibaculum* spp. and consisting of filamentous, Gram-negative (determined with the crystal violet/iodine technique), nonmotile rods (by phase-contrast microscopy) were subcultured and cryopreserved at −80°C. DNA from revived cultures on marine agar was extracted on a QIAcube (Qiagen) utilizing a QIAamp DNA QIAcube minikit, following the manufacturer’s recommendations. Twenty-three sequencing libraries were generated with a Nextera DNA Flex library preparation kit (Illumina), following the manufacturer’s standard protocol. Each library was sequenced on a MiSeq system (Illumina) with a v3 flow cell and 300-bp paired-end chemistry.

The resulting numbers of reads per sample are listed in Table 1. Default parameters were used for all software unless otherwise stated. BBduk (from BBmap package v38.18) was used to remove Nextera DNA Flex adapter sequences and to perform quality trimming (using trimq=24 and minlen=150). Reads were assembled with SPAdes v3.15.3 (3) using the careful option. The reads were mapped back on the assemblies with BBmap (using maxindel=80, minid=0.95, ambiguous=toss, and killbadpairs=true), and error correction was subsequently performed with Pilon v1.24 (4). The assemblies were annotated with the NCBI Prokaryotic Genome Annotation Pipeline (PGAP) (5), and the species and genomovar of each isolate were determined by average nucleotide identity (ANI) analysis using fastANI v1.32 (6). A threshold of 96% ANI was used at the species level and 97.5% at the genomovar level (7). Assemblies with GenBank accession numbers GCA_001483385.1 and GCA_900239185.1 were used as references for Tenacibaculum finnmarkense genomovar *finnmarkense*, GenBank accession numbers GCA_900239485.1 and GCA_900239495.1 for Tenacibaculum finnmarkense genomovar *ulcerans*, and GenBank accession numbers GCA_900239455.1 and GCA_900239305.1 for Tenacibaculum dicentrarchi (Table 1).

**TABLE 1:** Assembly information for the draft genomes of 23 *Tenacibaculum* species isolates from lumpfish (*Cyclopterus lupus*)

Isolate	Species and genomovar^*a*^	Genome length (bp)	No. of reads^*b*^	Sequencing depth (×)^*c*^	GC content (%)	No. of contigs	*N*_50_ (bp)	GenBank accession no.	SRA accession no.
NVIO-11151	T. finnmarkense gv. *ulcerans*	2,912,685	634,728	54.2	30.9	63	341,205	GCA_021206215.1	SRR16845319
NVIO-11836	T. dicentrarchi	2,665,418	1,274,204	121.9	30.2	43	290,045	GCA_021206235.1	SRR16845318
NVIO-11837	T. dicentrarchi	2,718,782	1,016,758	93.3	30.2	47	299,843	GCA_021206175.1	SRR16845307
NVIB-0099	T. finnmarkense gv. *ulcerans*	2,769,811	581,146	49.3	31.1	306	15,852	GCA_021206185.1	SRR16845303
NVIB-0249	T. finnmarkense gv. *finnmarkense*	2,755,194	595,486	53.6	31.1	45	336,252	GCA_021206115.1	SRR16845302
NVIB-0461	T. dicentrarchi	2,645,329	656,214	62.7	30.2	41	267,012	GCA_021206125.1	SRR16845301
NVIB-0562	T. dicentrarchi	2,715,212	834,776	76.9	30.2	60	301,712	GCA_021206145.1	SRR16845300
NVIB-0714	T. finnmarkense gv. *ulcerans*	2,948,091	899,454	73.1	31.0	101	113,984	GCA_021206095.1	SRR16845299
NVIB-1038	T. dicentrarchi	2,729,260	890,344	78.2	30.1	60	268,855	GCA_021206065.1	SRR16845298
NVIB-1058	T. finnmarkense gv. *ulcerans*	2,978,337	869,586	67.4	30.8	107	264,193	GCA_021206045.1	SRR16845297
NVIB-1306	T. finnmarkense gv. *ulcerans*	3,011,088	737,008	58.3	30.9	74	261,018	GCA_021206035.1	SRR16845317
NVIB-1785	T. finnmarkense gv. *finnmarkense*	2,907,856	1,011,694	87.0	30.9	75	126,450	GCA_021206015.1	SRR16845316
NVIB-2771	T. dicentrarchi	2,708,961	783,294	71.9	30.2	39	439,048	GCA_021205995.1	SRR16845315
NVIB-2925	T. finnmarkense gv. *ulcerans*	2,847,587	892,166	77.2	30.9	76	239,559	GCA_021205955.1	SRR16845314
NVIB-3068	T. dicentrarchi	2,760,614	1,026,834	91.7	30.1	66	274,565	GCA_021205975.1	SRR16845313
NVIB-3688	T. dicentrarchi	2,762,784	1,118,844	100.4	30.1	67	268,943	GCA_021205915.1	SRR16845312
NVIB-3865	T. finnmarkense gv. *ulcerans*	2,925,085	719,972	59.6	30.9	62	365,686	GCA_021205935.1	SRR16845311
NVIB-4078	T. finnmarkense gv. *finnmarkense*	3,093,483	1,094,616	85.2	30.9	135	130,306	GCA_021205895.1	SRR16845310
NVIB-4084	T. finnmarkense gv. *ulcerans*	2,973,195	892,392	73.8	30.9	135	58,884	GCA_021205875.1	SRR16845309
NVIB-4330	T. dicentrarchi	2,706,007	1,061,242	96.8	30.2	65	410,240	GCA_021205835.1	SRR16845308
NVIB-4331	T. finnmarkense gv. *ulcerans*	2,881,546	559,816	46.0	30.8	111	256,579	GCA_021205845.1	SRR16845306
NVIB-4332	T. finnmarkense gv. *finnmarkense*	2,888,640	716,276	61.3	31.0	61	338,514	GCA_021205815.1	SRR16845305
NVIB-4333	T. dicentrarchi	2,733,905	1,023,684	93.9	30.1	47	231,759	GCA_021205795.1	SRR16845304

a The species and genomovar of each isolate were determined by ANI analysis using fastANI, with similarity thresholds of 95% for species and 97.5% for genomovar.

b Read count after quality control.

c Sequencing depths were calculated on reads mapped back on the assemblies.

### Data availability.

This whole-genome shotgun project has been deposited in DDBJ/ENA/GenBank as BioProject PRJNA777885, with accession numbers for each assembly as shown in Table 1. The raw sequencing reads have been deposited in the Sequence Read Archive (SRA) as shown in Table 1.
